# Detection of glycosaminoglycan loss in articular cartilage by fluorescence lifetime imaging

**DOI:** 10.1117/1.JBO.23.12.126002

**Published:** 2018-12-21

**Authors:** Xiangnan Zhou, Anne K. Haudenschild, Benjamin E. Sherlock, Jerry C. Hu, J. Kent Leach, Kyriacos A. Athanasiou, Laura Marcu

**Affiliations:** a University of California, Department of Biomedical Engineering, Davis, California, United States; b University of California, Department of Biomedical Engineering, Irvine, California, United States; c UC Davis Health, Department of Orthopaedic Surgery, Sacramento, California, United States

**Keywords:** fluorescence lifetime spectroscopy and imaging, osteoarthritis, cartilage, autofluorescence, glycosaminoglycan

## Abstract

Glycosaminoglycan (GAG) loss is an early marker of osteoarthritis, which is a clinical late stage disease that affects millions of people worldwide. The goal of our study was to evaluate the ability of a fiber-based fluorescence lifetime imaging (FLIm) technique to detect GAG loss in articular cartilage. Native bovine cartilage explants (n=20) were exposed to 0 (control), 0.5 (low), or 1  U/mL (high) concentrations of chondroitinase ABC (cABC) to create samples with different levels of GAG loss. FLIm assessment (excitation: 355 nm; detection: channel 1: 375 to 410 nm, channel 2: 450 to 485 nm, channel 3: 530 to 565 nm) was conducted on depth-resolved cross-sections of the cartilage sample. FLIm images, validated with histology, revealed that loss of GAG resulted in a decrease of fluorescence lifetime values in channel 2 (Δ=0.44  ns, p<0.05) and channel 3 (Δ=0.75  ns, p<0.01) compared to control samples (channel 2: 6.34 ns; channel 3: 5.22 ns). Fluorescence intensity ratio values were lower in channel 1 (37%, p<0.0001) and channel 2 (31% decrease, p<0.0001) and higher in channel 3 (23%, p<0.0001) relative to control samples. These results show that FLIm can detect the loss of GAG in articular cartilage and support further investigation into the feasibility of *in vivo* FLIm arthroscopy.

## 1 Introduction

Articular cartilage is the near frictionless tissue at the end of long bones that allows smooth movement of joints and absorbs shock. Cartilage degenerative diseases such as osteoarthritis (OA) are major causes of disability worldwide^1^ and have a substantial contribution to healthcare cost.^2^ This large economic burden will continue to increase as the population ages. Clinical OA is a late-stage condition for which disease-modifying opportunities are limited. However, OA typically develops over decades, offering a long window of time to potentially alter its course. As such, characterization of pre- or early-OA disease states will be critical to support a paradigm shift from palliation of late disease toward prevention.^3^ Unfortunately, early diagnosis of OA is still a challenging, unmet clinical need that must be addressed.

Cartilage extracellular matrix (ECM) is composed primarily of 15% to 30% glycosaminoglycan (GAG) (per dry weight), 50% to 75% collagen (per dry weight), and 70% to 80% water (per wet weight). The remaining balance of dry weight includes minor protein molecules and chondrocytes.^4^^,^^5^ The mechanical properties of articular cartilage are determined by the biochemical composition of the main tissue constituents, GAG and collagen, creating a structure–function relationship in which GAG resists compressive loading and collagen resists tensile loading within the tissue.^6^ During the early stages of OA, the changes in cartilage are clinically silent. No visual, functional, or mechanical alterations of articular cartilage appear detectable. Gradual GAG loss is the first observable indication of OA. Left untreated, it will lead to a “death spiral” of increasing matrix degradation and reduced biomechanical properties.^7^ As a result, normal loading increases matrix damage, leading to a positive feedback loop of cartilage damage, pain, and destruction. This deterioration continues until the cartilage completely loses its ability to withstand load and is worn away to expose subchondral bone.^8^ Therefore, the detection of GAG loss is critical for the early diagnosis of OA.

Imaging modalities are an integral part of the diagnosis of OA. Conventional imaging modalities like arthroscopic imaging, standard radiography, ultrasonography, and magnetic resonance imaging (MRI) are widely used in daily clinical diagnosis.^9^^–^^11^ Despite that, arthroscopy is primarily a qualitative assessment technique that falls short of the laboratory assessment standards of histopathology, biochemical analysis, and biomechanical testing. Both radiography and ultrasound fail to provide critical information regarding the biochemical composition of the cartilage ECM. Conventional MRI allows accurate assessment of both cartilage morphology and biochemical composition (collagen ultrastructure and GAG concentration) but still suffers from low clinical image resolution.^12^

Over the past decade, optical imaging modalities have been studied to evaluate the biochemical makeup and structural organization of cartilage and are being actively explored for utility in cartilage research and OA diagnosis. This includes laser scanning confocal microscopy,^13^ second harmonic generation microscopy, multiphoton microscopy,^14^ Fourier transform infrared imaging and spectroscopy,^15^ and Raman spectroscopy.^16^ Unfortunately, challenges, including the complexity and high cost of instrumentation and low data acquisition speed, hamper their clinical translation. Moreover, optical coherence tomography (OCT) allowing evaluation of cartilage microstructure associated with cartilage health has been used arthroscopically as a translational research tool for early diagnosis of OA.^17^^,^^18^ Still, OCT lacks the ability to provide biochemical information.

Therefore, there is a need for clinical compatible imaging modalities for *in vivo* assessment of biochemical composition related to cartilage health. Fiber-based time-resolved fluorescence lifetime imaging (FLIm) has the potential to address this need. FLIm with a lower-case m was used as abbreviation to distinguish our fiber-based technology from fluorescence lifetime microscopy (FLIM). Over the past decade, several groups have reported that FLIm may be used for disease diagnosis and tissue characterization with applications in numerous clinical areas, including oncology, cardiology, and ophthalmology.^19^ Multimodal instrumentation combining FLIm and ultrasound has also been used to monitor matrix composition and mechanical properties of engineered cartilage tissue during maturation.^20^^,^^21^ Recent advantages in FLIm devices using miniature fiber optics probes, along with fast electronics enabling fast data acquisition speed, analysis, and real-time display^22^ make this technique highly compatible with modern clinical arthroscopes. Hence, the goal of the present study was to investigate the feasibility of using FLIm for early detection of cartilage disease. Specifically, this study focused on (1) evaluation of fluorescence properties of native articular cartilage and their variation across different depth-resolved zones, (2) investigation of whether FLIm can detect the depletion of major cartilage biochemical constituents involved in development of OA, namely GAG, and (3) determining whether FLIm parameters can be used to infer the level of GAG loss in cartilage.

## 2 Materials and Methods

### 2.1 Cartilage Harvest

**Fig. 1:**
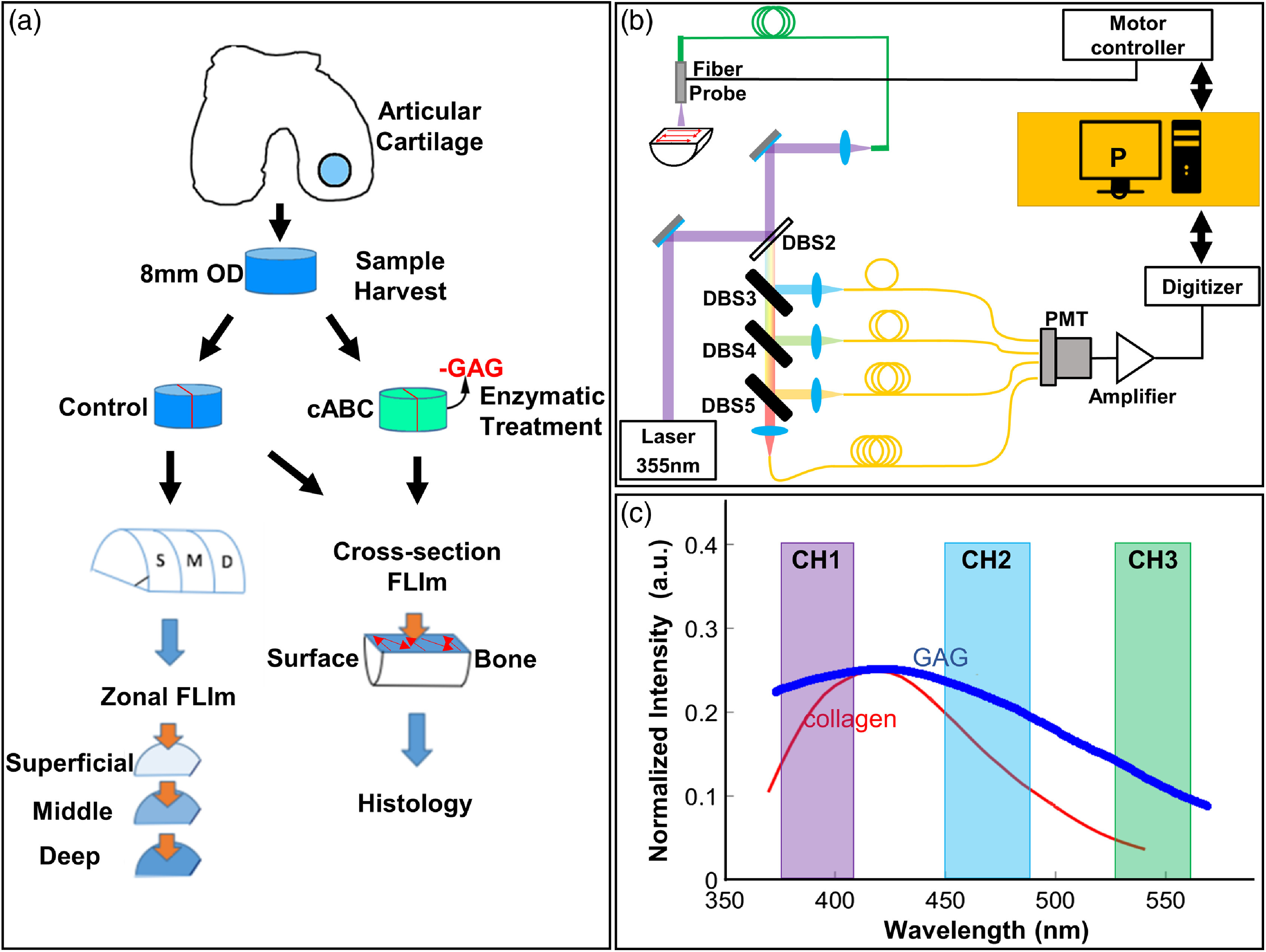
(a) Schematic diagram of the study protocol. Harvest and treatment of bovine articular cartilage samples in untreated (control) and cABC groups. Zonal en-face imaging was conducted on samples from different depths of cartilage from control group. Cross-sectional imaging was conducted on both control and cABC treatment groups. (b) Schematic diagram of the FLIm system. FLIm images were acquired by raster scanning of the optical fiber. (c) Spectral channels of the FLIm instrument: channel 1 = 375 to 410 nm, channel 2 = 450 to 485 nm, and channel 3 = 530 to 565 nm. The reference emission spectrum of collagen (red) and GAG (blue)^24^ was also overlaid.

Articular cartilage was excised as full thickness cylindrical osteochondral plugs (n=25) from a juvenile (2-week-old) bovine femoral head obtained from an abattoir (Research 87, Boylston, MA) within 48 h of death. Cartilage explants were wrapped in gauze and soaked in phosphate-buffered saline (PBS) with protease inhibitors (2-nM phenylmethylsulfonyl fluoride, 10-mM N-ethyl malemide, 2-mM ethylene diamine tetraacetatic acid, and 5-mM benzamidine-HCl) as previously described,^23^ underwent three freeze thaw cycles to lyse cells, and stored at −20°C in protease inhibitor until imaging [Fig. 1(a)].

### 2.2 Proteolytic Enzyme Treatments

Cartilage explants were placed one sample per well in six well plates (Costar, Corning, New York) in 6 mL of PBS (Sigma-Aldrich, St. Louis, Missouri). To remove proteoglycans, chondroitinase ABC (cABC) (Sigma-Aldrich) was added to a final concentration of 0  U/mL (control), 0.5  U/mL (low cABC), or 1  U/mL (high cABC) of cABC per well. All treatments included 50  U/mL salt activated nuclease (ArcticZymes, Tromsø, Norway), 0.02% ethylenediaminetetraacetic acid (Life Technologies, Grand Island, New York), and 1% penicillin/streptomycin/fungizone (Lonza, Basel, Switzerland). Treatment occurred for 10 h at 37°C on a rotatory plate at 35 revolutions/min. After treatment, explants were washed in fresh PBS for 12 h to remove residual enzymes.

### 2.3 FLIm Assessment of Articular Cartilage

After enzymatic treatment, full thickness cartilage samples were cut perpendicular to the surface into two half-cylinders. Samples were placed on a custom, glass sample holder for cross-sectional FLIm assessment. To study the depth-dependent fluorescence lifetime (LT) of cartilage, the other half of the cylinder was sectioned using a custom cutting jig into approximately the superficial, middle, and deep zones of the cartilage and imaged en-face [schematic is shown in Fig. 1(a)]. During FLIm acquisition, samples were immersed in PBS to maintain hydration. The fluorescence LT images were reconstructed from FLIm measurements and coregistered with histology images using the shape of the tissue sections. Regions of interest (ROIs) were selected in FLIm images following co-registration based on histology features for further analysis. Following imaging, cartilage samples were placed in formalin and processed routinely for histologic analysis. MATLAB (Mathworks, Inc.) software was used for image co-registration and ROIs selection.

### 2.4 FLIm Instrumentation

FLIm images were acquired using a prototype fiber-based point scanning principle and system, which had been previously reported.^25^^,^^26^ A schematic of the system is shown in Fig. 1(b). A frequency tripled Nd:YAG microchip laser (STV-02E-1x0, TEEM photonics) with repetition rate of 4K and pulse width of 600 ps was used as fluorescence excitation light source. Fluorescence excitation and emission were guided to and from the target by a single 400-μm core diameter all silica multimode fiber (FVP400440480, Polymicro). A wavelength selection module was used to separate fluorescence emission into three nonoverlapping spectral bands (channel 1 = 375 to 410 nm, channel 2 = 450 to 485 nm, channel 3 = 530 to 565 nm) [Fig. 1(c)]. The spectral channels were temporally separated by passing the emission pulse through three delay fibers (FVP400440480, Polymicro) of different lengths. A single microchannel plate photomultiplier tube (R3809U-50, Hamamatsu) detector was used to detect the temporally multiplexed signal. Neutral density (ND) filter with ND=1.3 was used to attenuated signals in detection channels 1 and 2 so all spectral channels had similar signal intensity. Raster scanning was achieved using a motorized three axis translation stage (MX80L, Parker) with scanning speed up to 500  mm/s. FLIm images were acquired with 200 or 400  μm square pixel size over a 10  mm×10  mm scanning area. The data acquisition time for each pixel was 8 ms, and the scanning speed was determined based on desired pixel size.

### 2.5 FLIm Parameters

Following the acquisition of the fluorescence decay signal, non-negative constrained least-square deconvolution based on the Laguerre expansion method was performed to determine the fluorescence response of cartilage samples.^27^ The average LT and intensity ratios were derived from the deconvolved fluorescence decay. The average LT was defined as the average amount of time a fluorophore stays in the excited state. Mathematically, it is the expected value of the probability distribution of detected photons, which was obtained by normalizing the deconvolved decay by the total area under the curve. Intensity ratios were defined as the ratio of fluorescence intensity at each channel to the sum of all three intensity channels. The standard deviation of the recovered LT was <0.1  ns.

### 2.6 Histology

After FLIm assessment, samples were fixed in 10% neutral buffered formalin, paraffin embedded, and then sectioned parallel to the imaging plane at 10  μm. The sections were stained with hematoxylin and eosin (H&E) for general morphology, safranin O for GAG, and picrosirius red (PSR) for total collagen following routine procedures. The sections were imaged using an inverted microscope (BZ-X700, Keyence).

### 2.7 Fluorescence Lifetime vs. Distance from Cartilage Surface

To study the variability of fluorescence LT inside the cartilage tissue, we studied the change of fluorescence LT as a function of distance from the cartilage surface. To determine the distance from the cartilage surface of each pixel, the location of cartilage surface in cross-sectional images was first determined by finding the pixels with maximum fluorescence emission intensity gradient. Those pixels were used as a reference point for distance calculation. After that, the distance to cartilage surface of every pixel inside cross-sectional LT images was calculated. A distribution of fluorescence LT at a given distance can be generated by combining data from all cross-sectional images (n=3).

### 2.8 Region of Interest Selection

ROIs were drawn manually based on the amount of safranin O staining in histology images. The GAG depleted region that had no or minimal safranin O staining was segmented manually as ROIs for data analysis. The thickness of each ROI, average value of fluorescence LT, and intensity ratio within each ROI were calculated and used for statistical analysis.

### 2.9 FLIm Image Segmentation

A simple threshold-based segmentation algorithm was developed and tested for its ability to detect GAG depletion in cross-sectional FLIm images. Channel 1 intensity ratio was used as the parameter for the segmentation algorithm and 0.25 was set as the threshold based on receiver operating characteristic analysis. The average depletion thickness was calculated and used for statistical analysis.

### 2.10 Statistics

In this study, n=25 biopsy samples were harvested from the same joint. We used n=3 to study the depth-dependent LT of native cartilage. We used the remaining n=22 (control = 7, low cABC=8, high cABC=7) to study the effect of GAG loss on cartilage fluorescence properties. Two samples were excluded from data analysis due to improper histology sectioning, resulted in the final total sample number of n=20 (control: n=6, low cABC: n=8, high cABC: n=6). Statistical analysis was performed using Student’s t-tests and one-way ANOVA with Tukey’s posthoc analysis, where applicable. All of the statistical analysis was performed with R Statistical Software (Foundation for Statistical Computing, Vienna), and p<0.05 were considered statistically significant.

## 3 Results

### 3.1 Depth-Dependent Lifetime of Native Articular Cartilage

**Fig. 2:**
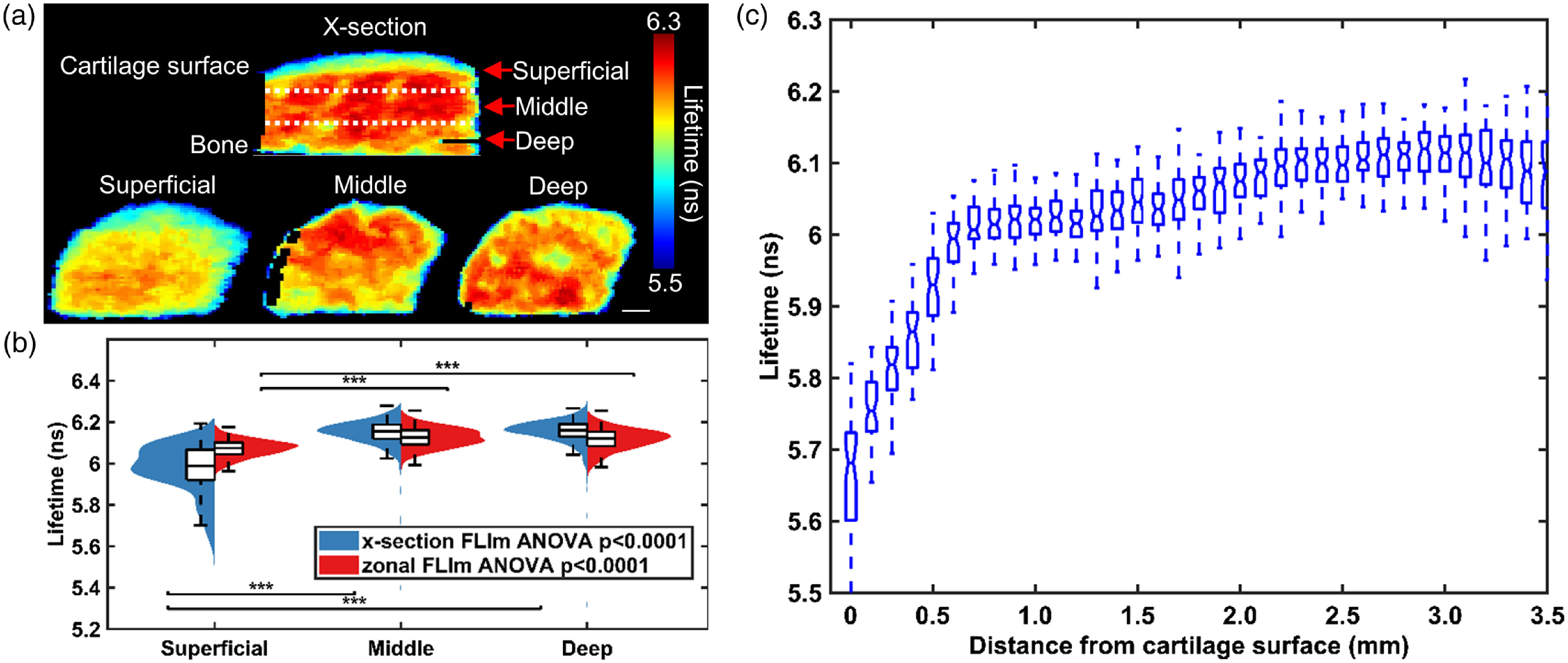
Channel 1 (375 to 410 nm) fluorescence LT increases with distance from articular cartilage surface (n=3). (a) Representative cross-section and zonal FLIm image of native bovine articular cartilage. Scalebar=1  mm. Dashed line represents estimated location of cut of superficial, middle and deep zone. (b) Violin plot of LT distribution of different zones of articular cartilage acquired through zonal and x-section imaging. The LT of superficial zone is significantly lower than that of middle and deep zones (***p<0.001). (c) A box plot of average LT versus distance from cartilage surface for all samples (n=3). Each box represents all data points from all samples at the corresponding distance from cartilage surface. Five values are highlighted: the extremes, the upper and lower quantiles, and the median. A clear increase of LT with distance from cartilage surface is observed. The increase is most prominent within the first 0.5 mm of cartilage surface.

Cross-sectional FLIm images [Fig. 2(a)] showed that fluorescence LT in channel 1 varied with cartilage depth. The region closest to the cartilage surface had a LT of 5.7±0.12  ns while the middle and deep part of cartilage had a longer LT of 6.1±0.08  ns (p<0.0001). The same trend was also presented in the zonal FLIm images [Fig. 2(a)]. This was further confirmed by the violin plot as the distributions of LT from both cross-sectional and zonal imaging shifted to higher LT values in middle and deep zone [Fig. 2(b)]. This trend was observed for all samples as depicted by the box-plot of LT distribution from all samples (n=3), which demonstrated that the LT increases with distance from cartilage surface. This increase was most noticeable within 500  μm from the surface [Fig. 2(c)]. In addition, changes of fluorescence LT were also associated with morphological heterogeneities. Macrostructures with shorter LT were also presented in both cross-section and en-face FLIm LT images [Fig. 2(a)]. No significant depth-dependent LT changes were observed in other spectral channels.

### 3.2 Detection of GAG-Depletion using FLIm

**Fig. 3:**
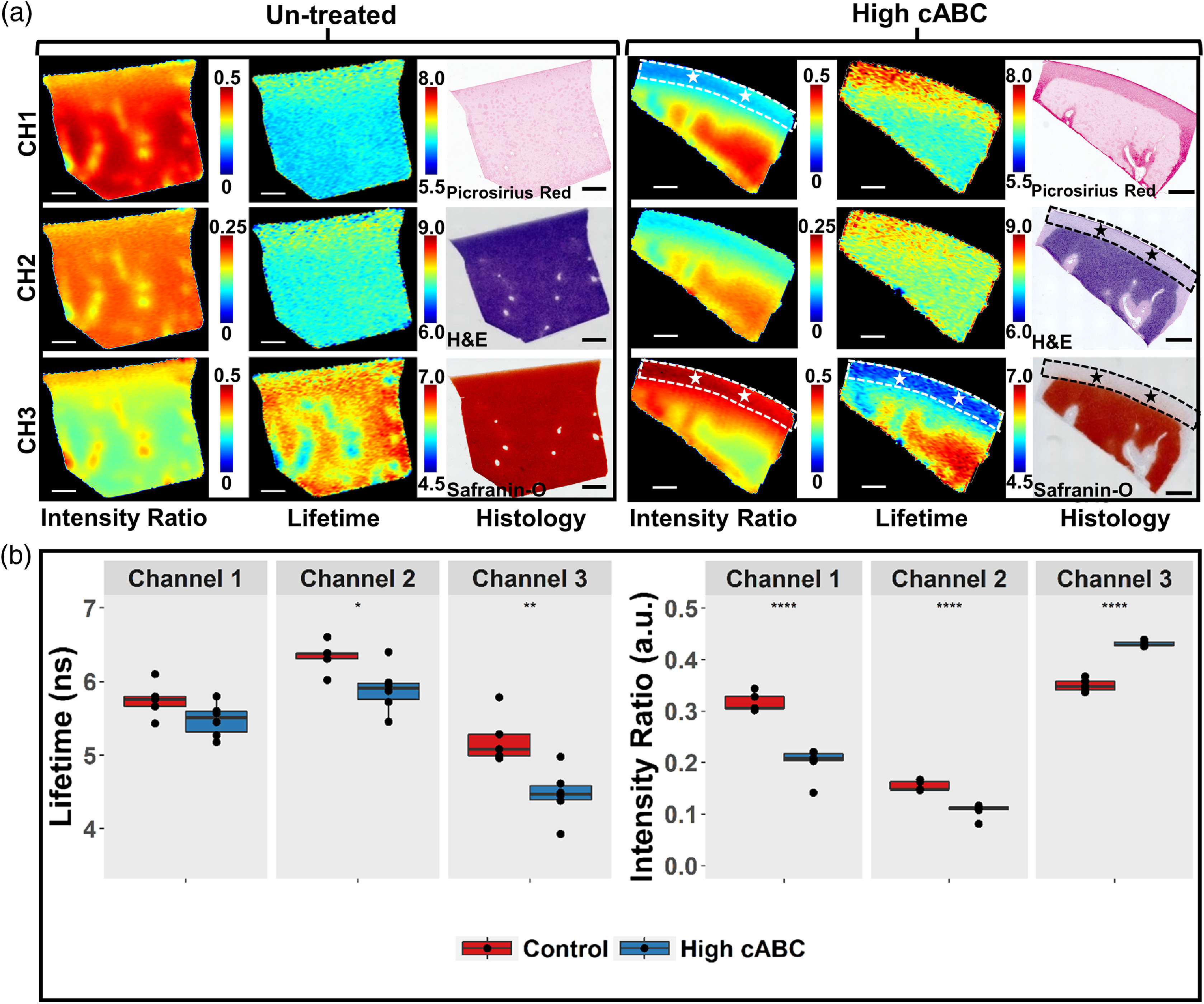
FLIm detects GAG depletion in articular cartilage. (a) Representative fluorescence cross-sectional intensity ratio images, LT images, and histology images of untreated and treated bovine articular cartilage. ROIs highlight the region of GAG depletion in histology images of cABC treated sample. The corresponding region in FLIm LT and intensity ratio images are highlighted by stars. The GAG depletion region appears in FLIm maps with higher intensity ratio and lower LT in channel 3 as well as lower intensity ratio in channel 1. No GAG depletion is observed for control sample. Scalebar=1  mm (n≥6 per group). (b) Box plot of ROI average LT from all samples in control and high cABC group. Significant LT differences are observed in channel 2 (450 to 485 nm) *p<0.05 and channel 3 (530 to 565 nm) **p<0.01. Significant differences in intensity ratio are observed in all channels. ****p<0.0001.

Safranin-O stained histology images showed that compared to untreated (control) cartilage, enzymatic treatment of cABC almost completely depleted GAG content within 1 mm distance from cartilage surface [Fig. 3(a)]. The depletion of GAG resulted in significant decreases in fluorescence LT in both detection channel 2 (p<0.05) and channel 3 (p<0.01) and significant changes in the intensity ratios in all collection channels (p<0.0001) [Fig. 3(b)].

**Fig. 4:**
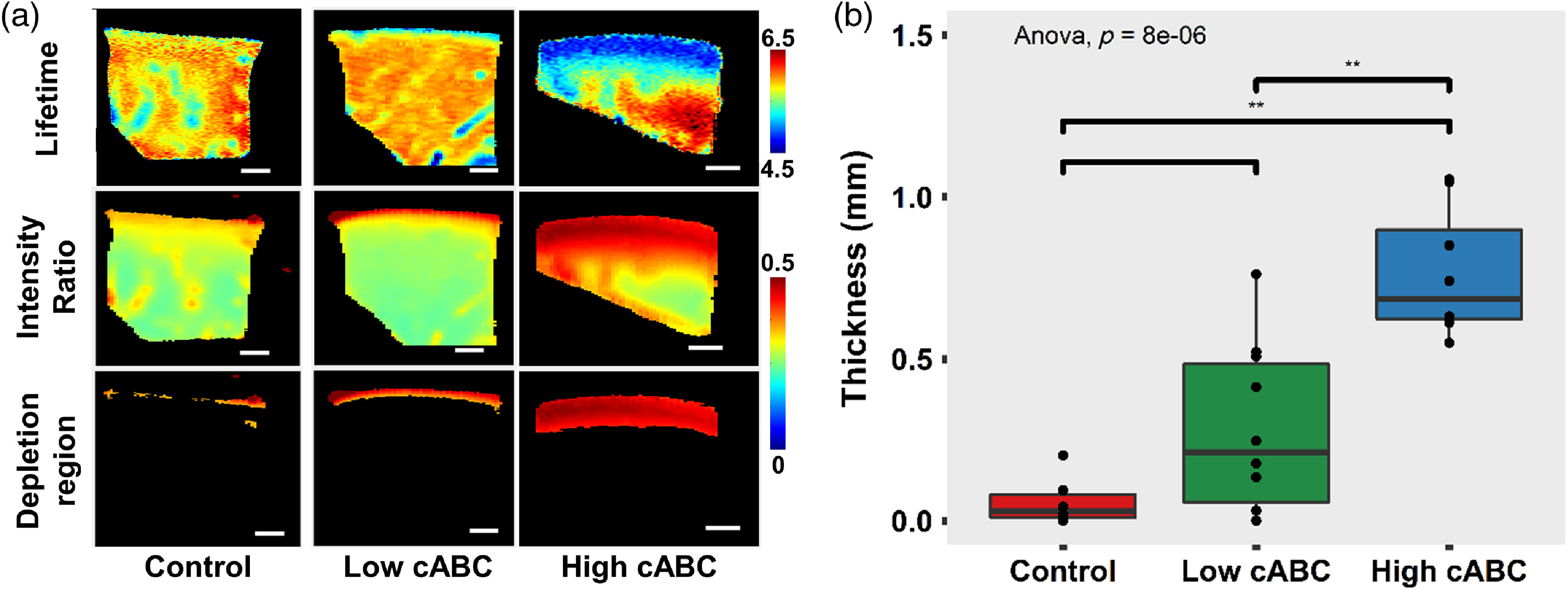
(a) Representative cross-sectional FLIm LT and intensity ratio images of cartilage without treatment and cartilage treated with high (2  U/mL) and low (1  U/mL) concentration of cABC. The depleted region is segmented using channel 1 intensity ratio of 0.25. Scalebar=1  mm. (b) Box plot of the thickness of the GAG depleted layer calculated from segmentation result **p<0.01 (n≥6 per treatment group).

A segmentation algorithm based on channel 1 intensity ratio was able to identify regions of GAG depletion in both low and high cABC treated FLIm images [Fig. 4(a)]. The untreated control group had an average depleted layer thickness of ∼0.1  mm, whereas low (1  U/mL) and high (2  U/mL) C-ABC concentration treatment resulted in an average depletion layer thickness of 0.3 mm and 0.7 mm, respectively. Average depletion thickness from high cABC treatment group was significantly higher than that of control (untreated) (p<0.01) and low cABC (p<0.01) treatment group. However, no significant difference was observed in average depletion region thickness between control and low cABC treatment group [Fig. 4(b)].

## 4 Discussion

This study demonstrated that (1) fluorescence LT of articular cartilage varies across its depth; shorter LT values were observed for the superficial zone of cartilage when compared with the middle and deep zones and (2) the depletion of GAG can be detected using FLIm and the level of GAG depletion in articular cartilage can be inferred using fluorescence-based parameters. Both of the above points demonstrate that FLIm can detect biochemical changes in cartilage tissue and has great potential for early detection of cartilage disease.

To our knowledge, this is the first study to map the fluorescence features of native bovine articular cartilage across its full thickness. The cross-sectional imaging protocol allowed us to study changes in FLIm parameters as a function of depth. The comparison of LT distribution obtained from cross-sectional imaging and that from conventional en-face imaging cross-validated the results. Given the complex zonal variation of cell morphology, collagen fiber orientation, and biochemical composition in articular cartilage,^8^ the change of fluorescence LT across the thickness of cartilage is expected. Current results are in agreement with earlier studies conducted on articular cartilage that showed shorter LT for the superficial layer relative to middle and deep zone of the cartilage^28^ and a previous study that validated en-face FLIm parameters with biochemical assays.^29^ These results, in conjunction with the fiber-based platform, allow for ease of translation into imaging in humans via augmentation of the current clinical practice of arthroscopy. By incorporating the optical fiber into a conventional viewing scope, this technique would enable straightforward visual evaluation of the integrity of the cartilage surface and inner structures, allow spatial or temporal monitoring during disease development, and allow clinicians to monitor the biochemical changes occurring within the patient.

Apart from the change of fluorescence LT as a function of depth, macrostructures presented in both cross-section (shape of tunnel) and en-face (round shape) exhibit shorter LT when compared with the surrounding cartilage mass. Since cartilage from juvenile bovine condyles was used in this study, these microstructures could be cartilage canals that present only in the early stage of cartilage development.^30^ Since cartilage canals have different biochemical composition when compared with the collagen-rich cartilage ECM, it is expected that these microstructures exhibit a different LT. This demonstrated that FLIm could be a potential tool for studying basic biochemical properties associated with structural heterogeneities of cartilage as well. To date, much is known about articular cartilage organization and ECM, but less is known about its developmental biology. How articular cartilage grows and matures into a complex, functional, and multifaced structure is still unclear. FLIm could help advance our knowledge in these research areas by monitoring the change of biochemical properties of native articular cartilage during its development and along its depth. The advances will have significant impact for basic science and potential translational value to the design of superior cartilage regeneration and rapier strategies.

The finding that the depletion of GAG significantly altered the fluorescence features of articular cartilage tissue and the change could be detected using our FLIm instrumentation is of critical importance for the research of OA diagnosis, as GAG loss is the first indicator for early OA development. This could enable early diagnosis of OA before clinical symptoms like pain and the appearance of cartilage loss.

We acknowledge that the freeze-thaw cycles of the cartilage tissue before imaging lyse the cells and could alter cartilage fluorescence properties. Thus, the experimental conditions reported in this study might not fully mimic conditions encountered in the clinical application. In a separate study conducted on articular cartilage using the same FLIm instrumentation, we showed that the freezing process results in decreases in channel 1 LT (Δ=0.14; p=0.003) and significant increases in channel 2 LT (Δ=0.11; p=0.003) and channel 3 LT (Δ=0.22; p=0.003) compared to fresh tissue. However, the changes were small compared to those induced by the loss of GAG, which resulted in a significant decrease of fluorescence LT values in channel 2 (Δ=0.44  ns; p<0.05) and channel 3 (Δ=0.75  ns; p<0.01). The small differences in FLIm LT values with cell lysis could be attributed to the very low (∼10% by volume) composition of cells within cartilage tissue.

Interestingly, collagen was more densely packed in the region of GAG depletion post-cABC treatment. This was represented in PSR stained histology as an increase in color saturation in the ROIs. In healthy cartilage, the collagen network is slightly stretched due to the Donnan osmotic pressure caused by GAG content that provides its compressive stiffness.^8^ Thus, the loss of GAG would lead to a slightly denser collagen matrix.

GAG loss resulted in a significant decrease in the intensity ratio of channels 1 and 2, which represents collagen and a significant increase in the intensity ratio of channel 3, which represents GAG. This nonintuitive change could be attributed to the conformational change of collagen structure inside cartilage tissue. It is well known that collagen cross-links strongly fluoresce. The loss of GAG could result in the breakdown of collagen cross-links, which was not quantified in this study. Thus, the loss of GAG very likely resulted in the loss of absolute intensity in all spectral channels, but the loss in channels 1 and 2 was more prominent due to the high quantum yield of collagen and its cross-links. As intensity ratio characterizes the relative intensity contribution of each channel instead of absolute intensity, a higher absolute loss in channels 1 and 2 will result in a decrease in channel 1 and channel 2 intensity ratios and a higher channel 3 intensity ratio.

The decrease of fluorescence LT in channel 2 and channel 3 is a result of the intensity ratio change. Collagen is the dominant fluorophore in cartilage and has the highest fluorescence LT (∼5.5  ns) of the major constituents.^21^ The decrease of intensity ratio in channel 1 and channel 2 indicates a lower contribution from collagen, which would result in a significant decrease of fluorescence LT in all channels. There is no significant change of fluorescence LT in channel 1 as collagen fluorescence completely dominates this channel. Channel 3 had the largest LT decrease as it sits at the tail of collagen emission spectrum and the collagen contribution is less dominant.

A threshold-based segmentation algorithm could distinguish high levels of GAG depletion in our cABC-treatment group from the control group using one of the many fluorescence parameters. However, intensity measurement is highly sensitive to the excitation and collection geometry. Any change of excitation and collection geometry could lead to significant measurement error. On the other hand, fluorescence LT is robust to the change of measurement conditions. Thus, the effectiveness and robustness of our instrumentation to detect early OA could be further improved by including more FLIm parameters. Nonetheless, we demonstrated that parameters from noninvasive FLIm imaging can successfully detect GAG depletion in articular cartilage.

We employed raster scanning by a translation stage in this study to automate the scanning process. In a clinical setting, the fiber-based interface can be introduced through a standard clinical arthroscope with the FLIm imaging data overlaid on the arthroscope image on the surgical monitor. We have previously demonstrated this concept using a hand scanning probe^31^ for interrogation of breast cancer tumors. Real-time FLIm data were overlaid on conventional white light images of the tissue,^32^ providing valuable real time feedback to the surgeons. When combined with machine learning algorithms, real-time classification was also possible, providing potential real-time disease discrimination.^31^

We acknowledge that the cross-section imaging approach used in this study is different from an arthroscopy imaging situation. Future en-face imaging will be required to properly assess the performance of FLIm instrumentation for detection of OA and its usefulness in clinical environment. Furthermore, we acknowledge that FLIm is only a surface imaging modality with a penetration depth of ∼300  μm and is not able to provide any structural information regarding the integrity of articular cartilage. However, FLIm can be adapted to tissue interrogation in endoscopic-like configurations,^33^^,^^34^ thus, it can complement other structural imaging modalities like ultrasound and OCT. This study serves as an important step toward early diagnosis of OA and encourages further investigation into the feasibility of *in vivo* FLIm arthroscopy.

## 5 Conclusion

This study demonstrates that GAG degradation in articular cartilage can be detected by rapid, nondestructive, fiber-based FLIm. This technique could be utilized as a potential tool for basic research of cartilage. In addition, the size of the miniature fiber probe, the clinically compatible high imaging speed, and the potential for multimode imaging, such as FLIm-OCT,^35^ make it a promising tool for the early diagnosis of OA. This technique has a great potential to facilitate a paradigm shift from palliation of late disease toward OA prevention and early intervention.
